# Survival and prognosis with osteosarcoma: outcomes in more than 2000 patients in the EURAMOS-1 (European and American Osteosarcoma Study) cohort

**DOI:** 10.1016/j.ejca.2018.11.027

**Published:** 2019-03

**Authors:** Sigbjørn Smeland, Stefan S. Bielack, Jeremy Whelan, Mark Bernstein, Pancras Hogendoorn, Mark D. Krailo, Richard Gorlick, Katherine A. Janeway, Fiona C. Ingleby, Jakob Anninga, Imre Antal, Carola Arndt, Ken L.B. Brown, Trude Butterfass-Bahloul, Gabriele Calaminus, Michael Capra, Catharina Dhooge, Mikael Eriksson, Adrienne M. Flanagan, Godehard Friedel, Mark C. Gebhardt, Hans Gelderblom, Robert Goldsby, Holcombe E. Grier, Robert Grimer, Douglas S. Hawkins, Stefanie Hecker-Nolting, Kirsten Sundby Hall, Michael S. Isakoff, Gordana Jovic, Thomas Kühne, Leo Kager, Thekla von Kalle, Edita Kabickova, Susanna Lang, Ching C. Lau, Patrick J. Leavey, Stephen L. Lessnick, Leo Mascarenhas, Regine Mayer-Steinacker, Paul A. Meyers, Raj Nagarajan, R.Lor Randall, Peter Reichardt, Marleen Renard, Catherine Rechnitzer, Cindy L. Schwartz, Sandra Strauss, Lisa Teot, Beate Timmermann, Matthew R. Sydes, Neyssa Marina

**Affiliations:** a SSG Oslo University Hospital and Scandinavian Sarcoma Group and Institute for Clinical Medicine, University of Oslo, Norway; b COSS Klinikum Stuttgart - Olgahospital Stuttgart, Germany; c EOI University College Hospital, London, UK; d COG IWK Health Center, Dalhousie University, Halifax, NS, Canada; e TMG Path Leiden University Medical Centre, Leiden, Netherlands; f COG Children’s Oncology Group, Arcadia, CA, USA; g COG the University of Texas M D Anderson Cancer Center, Houston, TX, USA; h COG Dana-Farber Cancer Institute, Boston, MA, USA; i CDC MRC Clinical Trials Unit at UCL, London, UK; j EOI, Netherlands; k COSS Semmelweis Egyetem Budapest, Budapest, Hungary; l COG Mayo Clinic, Rochester, MN, USA; m COG University of British Columbia, Vancouver, BC, Canada; n EISD Centre for Clinical Trials, University Hospital Muenster, Muenster, Germany; o QLCC Pädiatrische Hämatologie und Onkologie, Universitätsklinikum Bonn, Bonn, Germany; p EOI Our Lady's Children's Hospital, Dublin, Ireland; q EOI University Hospital Ghent, Gent, Belgium; r SSG Lund University, Lund, Sweden; s EOI Royal National Orthopaedic Hospital, Stanmore; Cancer Institute, University College London, London, UK; t COSS Klinik Schillerhöhe – Thoraxchirurgie Gerlingen, Germany; u EOI Leiden University Medical Center, Leiden, the Netherlands; v COG UCSF Medical Center-Mission Bay, Pediatric Oncology, San Francisco, CA, USA; w EOI Royal Orthopaedic Hospital, Birmingham, UK; x COG University of Washington, Seattle, WA, USA; y COSS Universitätsklinikum Essen, Essen, Germany; z COG Five Prime Therapeutics, Inc South San Francisco, CA, USA; aa SSG Oslo University Hospital, Oslo, Norway; ab COG Connecticut Children’s Medical Center, Hartford, CT, USA; ac COSS Universitätsspital Basel, Basel, Switzerland; ad COSS St. Anna Kinderspital /CCRI, Wien, Austria; ae COSS University Hospital MOTOL, Praha, Czech Republic; af COSS Medizinische Universität Wien, Vienna, Austria; ag COG Baylor College of Medicine, Houston, TX, USA; ah COG Southwestern and Children’s Medical Center, Dallas, TX, USA; ai COG Nationwide Children’s Hospital and the Ohio State University, Columbus, OH, USA; aj COG Keck School of Medicine, University of Southern California, Los Angeles, CA, USA; ak COSS Universitätsklinikum Ulm, Ulm, Germany; al COG Memorial Sloan Kettering Cancer Center, New York, NY, USA; am COG Cincinnati Children’s Hospital Medical Center, Cincinnati, OH, USA; an COG Primary Childrens Hospital, The University of Utah, Salt Lake City, UT, USA; ao COSS Helios Kliniken Berlin-Buch, Berlin, Germany; ap EOI University Hospital Leuven, Leuven, Belgium; aq SSG Rigshospitalet, University of Copenhagen, Copenhagen, Denmark; ar COG Boston Children’s Hospital, Boston, MA, USA

**Keywords:** Osteosarcoma, Chemotherapy, Surgery, Cohort, Outcomes

## Abstract

**Background:**

High-grade osteosarcoma is a primary malignant bone tumour mainly affecting children and young adults. The European and American Osteosarcoma Study (EURAMOS)-1 is a collaboration of four study groups aiming to improve outcomes of this rare disease by facilitating randomised controlled trials.

**Methods:**

Patients eligible for EURAMOS-1 were aged ≤40 years with M0 or M1 skeletal high-grade osteosarcoma in which case complete surgical resection at all sites was deemed to be possible. A three-drug combination with methotrexate, doxorubicin and cisplatin was defined as standard chemotherapy, and between April 2005 and June 2011, 2260 patients were registered. We report survival outcomes and prognostic factors in the full cohort of registered patients.

**Results:**

For all registered patients at a median follow-up of 54 months (interquartile range: 38–73) from biopsy, 3-year and 5-year event-free survival were 59% (95% confidence interval [CI]: 57–61%) and 54% (95% CI: 52–56%), respectively. Multivariate analyses showed that the most adverse factors at diagnosis were pulmonary metastases (hazard ratio [HR] = 2.34, 95% CI: 1.95–2.81), non-pulmonary metastases (HR = 1.94, 95% CI: 1.38–2.73) or an axial skeleton tumour site (HR = 1.53, 95% CI: 1.10–2.13). The histological subtypes telangiectatic (HR = 0.52, 95% CI: 0.33–0.80) and unspecified conventional (HR = 0.67, 95% CI: 0.52–0.88) were associated with a favourable prognosis compared with chondroblastic subtype. The 3-year and 5-year overall survival from biopsy were 79% (95% CI: 77–81%) and 71% (95% CI: 68–73%), respectively. For patients with localised disease at presentation and in complete remission after surgery, having a poor histological response was associated with worse outcome after surgery (HR = 2.13, 95% CI: 1.76–2.58). In radically operated patients, there was no good evidence that axial tumour site was associated with worse outcome.

**Conclusions:**

In conclusion, data from >2000 patients registered to EURAMOS-1 demonstrated survival rates in concordance with institution- or group-level osteosarcoma trials. Further efforts are required to drive improvements for patients who can be identified to be at higher risk of adverse outcome. This trial reaffirms known prognostic factors, and owing to the large numbers of patients registered, it sheds light on some additional factors to consider.

## Highlights

• Osteosarcoma is a rare disease, and treatment can only improve with international collaboration.
• We have assembled prospectively collected data from treatments of all the patients in the intercontinental European and American Osteosarcoma Study-1 protocol.
• These consistently treated patients provided a strong data set for reporting survival outcomes and reporting on prognostic factors.
• The trial reaffirms known prognostic factors, and the most adverse factors were metastases and tumours in the axial skeleton.
• Owing to the large numbers of patients registered, light is shed on some additional factors to be considered. Around seven in ten patients were still alive five years after diagnosis.

## 1 Introduction

Osteosarcoma is a malignant bone tumour mainly affecting children and young adults. Although osteosarcoma is the most common primary malignant bone cancer, it is a rare disease and has an annual incidence of 3–4 patients per million. The introduction of multi-agent chemotherapy several decades ago improved 5-year event-free survival in localised high-grade osteosarcoma from less than 20% to around 60%. Since then, there have been few evidence-based improvements introduced shown to improve survival [1], [2], [3], [4]. The European and American Osteosarcoma Study (EURAMOS) collaboration, initiated by four internationally recognised study groups, was formed to improve outcomes in osteosarcoma by facilitating the conduct of randomised controlled trials (RCTs) [5]. These groups were the Children's Oncology Group (COG), Cooperative German-Austrian-Swiss Osteosarcoma Study Group (COSS), European Osteosarcoma Intergroup (EOI) and Scandinavian Sarcoma Group (SSG).

The EURAMOS-1 trial was a risk-stratified randimised controlled trial, investigating treatment optimisation on the basis of histological response to pre-operative chemotherapy. Patients eligible for EURAMOS-1 were aged ≤40 years at diagnosis with localised or metastatic skeletal osteosarcoma in which case complete surgical resection at all sites was deemed to be possible. The extensive international collaboration enabled more rapid accrual than any trial groups could have achieved alone; from April 2005 to June 2011, 2260 patients were registered (enrolled) to the protocol [6].

The EURAMOS-1 collaboration agreed on a standard of care for osteosarcoma chemotherapy, in which there had been various approaches used. Accordingly, the three-drug combination with methotrexate, doxorubicin and cisplatin following the previous COG trial was defined as standard chemotherapy [7], [8]. Thus, the study cohort represents a large number of patients uniformly treated according to the same protocol.

The key adverse prognostic factors at presentation for survival in osteosarcoma are presence of metastases, large tumour volume and non-extremity (axial) site of the primary tumour. After surgical resection, response to pre-operative chemotherapy and achievement of surgical remission status are prognostically important [9], [10], [11], [12], [13], [14].

Of the 2260 registered patients for EURAMOS-1, 1334 (59%) joined one of the two randomisations [6]. The results of the trial have been previously reported: No evidence was found that either research treatment improved event-free survival, the primary outcome measure [15], [16]. The aim of these further analyses is to report outcomes for the whole cohort of eligible registered patients, as timed from diagnostic biopsy. We consider the prognostic impact of factors measured at diagnosis and the impact of response to pre-operative chemotherapy in patients with initially localised disease, timed from surgery.

## 2 Methods

### 2.1 Patient selection

The EURAMOS-1 protocol contains two open-label randomised phase III comparisons for patients with high-grade osteosarcoma, split by good and poor histological response to pre-operative chemotherapy, embedded within one overall patient cohort including all those registered/enrolled in the trial. The trial structure, eligibility criteria and patient assessments have been described previously [5], [6]. Patients aged ≤40 years with newly diagnosed osteosarcoma could be registered within 30 days after the diagnostic biopsy. Diagnostic biopsies were examined by an institutional pathologist and reviewed by each study group's reference pathologist. Patients with high-grade localised or metastatic, extremity or axial osteosarcoma deemed to be resec*table* by their treating team were potentially eligible pending specific criteria. These included adequate performance status; cardiac, hearing, bone marrow, liver and renal function; no history of chemotherapy for previous malignancy and no prior treatment for osteosarcoma. Regulatory approval, ethics approval and consent were obtained according to national requirements before registration. Registration was preferred before treatment started but could be done up to 30 days afterwards.

All patients were planned for the same pre-operative therapy for 10 weeks consisting of 120 mg/m^2^ of cisplatin and 75 mg/m^2^ of doxorubicin (weeks 1 and 6) followed by 12 g/m^2^ of high-dose methotrexate (weeks 4, 5, 9 and 10). A subset of consenting patients meeting further eligibility criteria were randomised post-operatively based on histological response to pre-operative chemotherapy; overall, 1334 of 2260 (59%) registered patients were randomised [6].

**Fig. 1:**
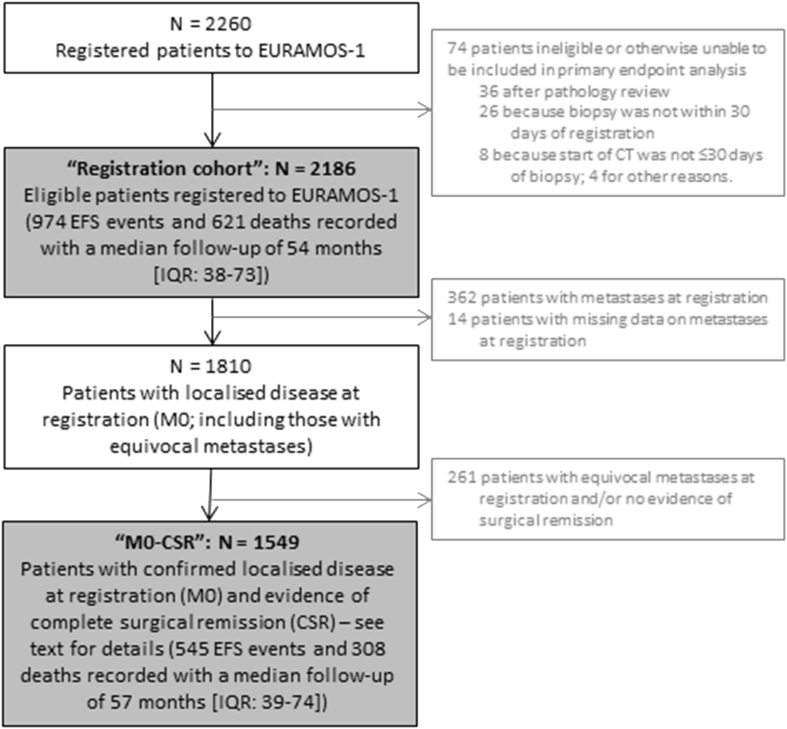
Flow diagram describing patient cohorts included in the ‘registration cohort’ and the ‘M0-CSR’ groups for analysis. CT, computed tomography; EFS, event-free survival; IQR, interquartile range; EURAMOS-1, European and American Osteosarcoma Study-1.

The aim of the present analysis was to report patient outcomes in two key populations: from biopsy, the full ‘registration cohort’, including all registered patients and excluding any ineligible patients, i.e. those who could be included in the primary end-point analysis; and from surgery, the ‘M0-CSR’ subgroup, which was the subset of the ‘registration cohort’ without baseline metastases and who achieved complete surgical remission (CSR). Surgical remission and margins were taken as reported by the surgeon and pathologist, respectively. We also present outcome data by metastatic status for patients in the ‘registration cohort’. Details are given in Fig. 1.

We required measures to consistently define non-metastatic (M0) and metastatic (M1) disease in patients across the four trials groups. The COSS, EOI and SSG categorised metastases as ‘no’, ‘possible’ and ‘yes’, whereas COG used only ‘no’ and ‘yes’. To reflect this difference, we grouped together patients with ‘no’ and ‘possible’ metastases as M0 patients, distinct from M1 patients with confirmed metastases by imaging criteria. Patients registered with ‘possible’ metastases, who later record a first event of ‘progression of existing metastases’ rather than ‘new metastases’, were retrospectively reclassified as M1 patients at registration because this ensured that the reporting of progression events was consistent with patient data at registration; the site must already have made this decision.

The ‘M0-CSR’ group of patients primarily includes patients for whom surgical remission or macroscopic clearance was explicitly reported on the case report form by the surgeon. However, one or both of these data items were missing for some patients, mostly patients who were registered but not randomised for whom the protocol permitted a reduced burden of form completion. If one of these items was reported and the other was missing, the patient was included in the ‘M0-CSR’ group, and providing this information was consistent with the disease status on the first, timely follow-up form. If both data items were missing, the patient was included in the ‘M0-CSR’ group only if the first post-treatment follow-up form stated that the patient was in remission within 1 year of the surgery date. Patients were excluded from the ‘M0-CSR’ group if they had reported an event-free survival (EFS) event before surgery.

### 2.2 Outcome measures

The primary outcome measure was EFS, defined as the time to first event (local recurrence, new metastases, progression of existing metastases, second malignancy, death or a combination of those events) or censoring at last contact. The first event was changed to ‘local progression’ where sites reported ‘local recurrence’ before or without surgery (this applied to 62 patients). Overall survival was defined as the time to death or censoring at last contact. The start time for assessing EFS and survival varies according to the analysis: EFS and survival were timed from the date of diagnostic biopsy for the ‘registration cohort’ and from the date of surgery of the primary tumour (i.e. a landmark approach) for the ‘M0-CSR cohort’.

### 2.3 Sample size

The sample size calculation for the original trial was based on the number required for each of the two separate, post-operative randomisations: 567 good response and 693 poor response patients (N = 1260 total). These, in turn, were driven by the number of EFS events required for the design parameters (as mentioned previously). To randomise 1260 patients, it was initially planned to register around 1400 patients, but because the randomisation rate was lower than that anticipated, the sample size for registration was increased to around 2000 registrations. Therefore, the size of the registered but not randomised patients was substantially larger here than originally envisaged.

Detailed data on surgery and post-operative chemotherapy were not collected for patients who were registered to the trial but who were not randomised because our main focus was outcomes in randomised comparisons. Follow-up was expected for all registered patients according to the previously described schedules.

### 2.4 Statistical analyses

Survival curves were estimated using the Kaplan–Meier method; Cox multivariate models, stratified by study group (COG, COSS, EOI and SSG), were applied. For all patients, the following variables were included in the multivariate models: tumour site and location within bone (proximal femur/humerus, other limb site or axial skeleton), pulmonary and non-pulmonary metastases status at registration, gender, pathological fracture at diagnosis, age, relative tumour volume (<1/3 or ≥1/3 of the involved bone), histological response to surgery, surgical margins as reported by the pathologist (wide/radical, marginal or intralesional) and World Health Organisation (WHO) classification of sarcoma. Conventional osteosarcomas were split into three groups after central review: osteoblastic, chondroblastic and other. Three age groups were defined according to Collins *et al.*: child (male: 0–12 years; female: 0–11 years), adolescent (male: 13–17 years; female: 12–16 years) and adult (male: 18 or older; female: age 17 years or older) [17]. Relative tumour size was the most commonly missing data item. To address this, we applied multiple imputations, creating 20 data sets with imputed tumour size data to cope with missingness of almost 20% of patients [18]. There was no evidence of a difference in survival with either previously reported research treatment; no analyses here are broken by allocated randomised treatment.

## 3 Results

### 3.1 Baseline characteristics

Overall, 2260 patients from 17 countries and 325 hospital sites were registered between April 2005 and June 2011 [6]. Seventy-four of these registered patients were either ineligible according to the trial eligibility criteria or unable to be included in the primary outcome analysis: 36 were ineligible after central pathology review (diagnosis other than high-grade skeletal osteosarcoma); 26 were registered later than 30 days after diagnostic biopsy; 8 did not start chemotherapy within 30 days after diagnostic biopsy and the remaining 4 patients were ineligible for other reasons. The remaining 2186 registered patients formed the ‘registration cohort’. In this cohort, median age at biopsy was 14 years (interquartile range [IQR]: 11–17), 59% (1285/2186) were male, 93% (1997/2138) had conventional osteosarcoma and 17% (362/2172) had metastases (Table 1). The primary tumour site was axial skeleton in 5% (106/2172), proximal femur or humerus in 13% (282/2172) and other limb site in 82% (1784/2172) of patients (Table 1).

**Table 1:** Characteristics at registration for all ‘registration cohort’ patients, split by metastatic status at registration (N = 2186).

Patient characteristic	M0^a^ at registration		M1 at registration		Metastases status at registration not known		Total	
	N	%	N	%	N	%	N	%
**Age at registration**^b^								
Child	536	30	115	32	6	43	657	30
Adolescent	900	50	168	46	7	50	1075	49
Adult	374	21	79	22	1	7	454	21
**Gender**								
Male	1050	58	230	64	5	36	1285	59
Female	760	42	132	36	9	64	901	41
**Site of the tumour**								
Proximal femur/humerus	227	13	55	15	0	0	282	13
Other limb site	1489	82	295	82	0	0	1784	82
Axial skeleton	94	5	12	3	0	0	106	5
*Missing*	*0*	*0*	*0*	*0*	*14*	*100*	*14*	*1*
**Location on the bone**								
Proximal	712	39	144	40	1	7	857	39
Diaphysis	73	4	19	5	0	0	92	4
Distal	917	51	184	51	0	0	1101	50
Not long bone, n/a	103	6	12	3	0	0	115	5
*Missing*	*5*	*0*	*3*	*1*	*13*	*93*	*21*	*1*
**WHO classification of sarcoma at diagnostic biopsy**								
Conventional: chondroblastic	303	17	39	11	1	7	343	16
Conventional: osteoblastic	1051	58	246	68	8	57	1305	60
Conventional: other	298	16	52	14	5	36	355	16
Telangiectatic	84	5	11	3	0	0	95	4
Small cell	9	1	2	1	0	0	11	1
High-grade surface	25	1	2	1	0	0	27	1
*Missing*	*40*	*2*	*10*	*3*	*0*	*0*	*50*	*2*
**Relative tumour volume**								
Small (<1/3 of involved bone)	848	47	110	30	1	7	959	44
Large (≥1/3 of involved bone)	638	35	177	49	0	0	815	37
*Missing*	*324*	*18*	*75*	*21*	*13*	*93*	*412*	*19*
**Pathological fracture at diagnosis**								
No	1594	88	308	85	1	7	1903	87
Yes	213	12	54	15	0	0	267	12
*Missing*	*3*	*0*	*0*	*0*	*13*	*93*	*16*	*1*
**Surgical margins achieved**^c^								
Wide/Radical	1357	75	257	71	13	93	1627	74
Marginal	218	21	34	9	0	0	252	12
Intralesional	23	1	3	1	0	0	26	1
*Missing*	*212*	*12*	*68*	*19*	*1*	*7*	*281*	*13*
**Duration of symptoms (weeks)**								
Median (IQR)	8		8		–		8	
Min–max	0–312		0–67		–		0–312	
N	1596		324		1^d^		1921	
**Total**	**1810**	**100**	**362**	**100**	**14**	**100**	**2186**	**100**

IQR, interquartile range; WHO, World Health Organisation.

a includes possible metastases.

b age groups defined according to Collins *et al.*: child (0–12 for males and 0–11 for females), adolescent (13–17 for males and 12–16 for females) and adult (≥18 for males and ≥17 for females).

c as reported by the pathologist.

d data not presented because duration of symptoms is known for only one patient in this group.

### 3.2 Outcomes from diagnosis (‘registration cohort’: N = 2186)

The ‘registration cohort’ patients had a median follow-up of 54 months (IQR: 38-73) from diagnostic biopsy, and 45% (974/2186) of patients reported an EFS event. Three-year EFS from biopsy was 59% (95% confidence interval [CI] 57–61%), and 5-year EFS was 54% (95% CI: 52–56%).

The breakdown of types of first event for the 974 ‘registration cohort’ patients reporting at least one EFS event is shown in Table 2.

**Table 2:** Summary of types of first event, as reported for the 974 ‘registration cohort’ patients in whom an EFS was reported.

Type of event	N	%
New metastases	521	53
Combination of events	144	15
*New metastases/progression of existing metastases*	*58*	*40*
*Local recurrence/new metastases*	*52*	*36*
*Local recurrence/new metastases/progression of existing metastases*	*13*	*9*
*Other or unknown combination*	*21*	*15*
Progression of existing metastatic disease	89	9
Local progression	74	8
Local recurrence	70	7
Death without any previously reported progression	41	4
*Cause of death attributed to osteosarcoma/treatment*^a^	*32*	*78*
*Other cause of death*	*9*	*22*
Secondary malignancy	26	3
Unknown event type	9	1
**Total**	**974**	**100**

a Or implicitly attributed to osteosarcoma/treatment as death occurred during therapy.

**Fig. 2:**
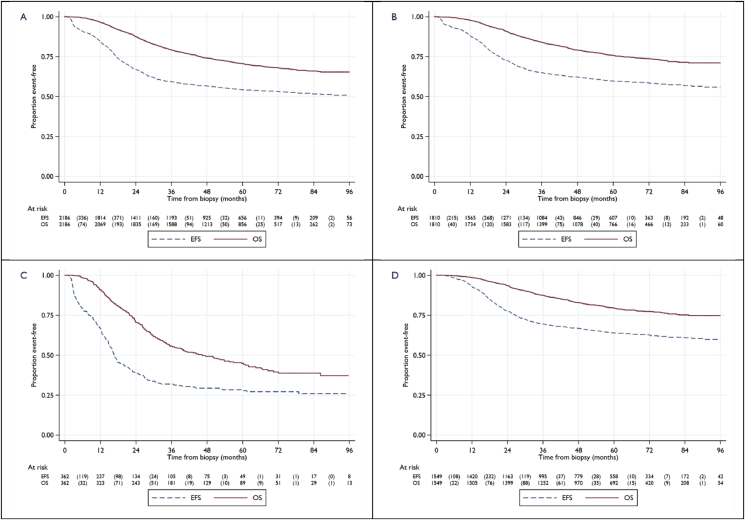
Kaplan–Meier plots for event-free survival and overall survival. (A) Full ‘registration cohort’. (B) Subset of ‘registration cohort’ patients with localised disease at registration (M0).M0 group includes patients with no metastases and possible metastases. (C) Subset of ‘registration cohort’ patients with metastatic disease at registration (M1). (D) ‘M0-CSR’ group, *Note that the number of patients at risk at the time of surgery is smaller for EFS than for OS because some patients had EFS event before surgery. EFS, event-free survival; OS, overall survival.

Note that the 41 patients in Table 2 with death as the first event without a previously reported progression event are a subset of the overall total of 621 deaths reported within the ‘registration cohort’. Out of these 621 deaths, 84% (524/621) of these were attributed to osteosarcoma; 3% (16/621) to treatment or during therapy (10 within a year of registration; 6 subsequently) and 6% (36/621) to other causes, including second malignancy. The cause of death is not reported for the remaining 7% (45/621). Three-year survival from biopsy was 79% (95% CI: 77–81%), and 5-year survival was 71% (95% CI: 68–73%) (Fig. 2A).

For multivariable analyses, 243 of 2186 patients were excluded due to missing data in multiple variables. Out of 412 patients with missing tumour size data, size was imputed for 336 patients. Based on a multivariate model of EFS with 1867 patients from the ‘registration cohort’ recording 762 EFS events (Table 3), poorer EFS was associated with having pulmonary metastases (HR = 2.34, 95% CI: 1.95–2.81) or non-pulmonary metastases (HR = 1.94, 95% CI: 1.38–2.73) at diagnosis, compared to having no metastases; having an axial skeleton tumour site (HR = 1.53, 95% CI: 1.10–2.13) or proximal femur/humerus tumour site (HR = 1.50, 95% CI: 1.22–1.84) compared to other limb site; being adult (HR = 1.32, 95% CI: 1.07–1.63) or adolescent (HR = 1.25, 95% CI: 1.05–1.48) compared to being a child; being male compared to female (HR = 1.20, 95% CI: 1.03–1.39) and having large relative tumour volume compared to small (HR = 1.29, 95% CI: 1.09–1.51). Improved EFS was associated with telangiectatic (HR = 0.52, 95% CI: 0.33–0.80), high-grade surface (HR 0.44, 95% CI: 0.19–0.99) and conventional unspecified subtype (HR = 0.67, 95% CI: 0.52–0.88) classifications, compared to chondroblastic. A model excluding the imputed tumour size data for 336 patients shows similar HRs as the model with the imputed data included. The CIs were broader in this model (due to fewer patients), but the interpretation is the same (Appendix Table 1).

**Table 3:** Cox model for event-free survival (timed from diagnostic biopsy) for all ‘registration cohort’ patients, *N = 1867*.

Characteristic	*N*	EFS events	Adjusted HR (95% CI)	*p*-value	Overall *p*-value
**Pulmonary metastases**					
No metastases^a^	1633	607	1.00	n/a	<0.001
Metastases	234	155	2.34 (1.95–2.81)	<0.001	
**Other metastases**					
No metastases	1809	724	1.00	n/a	<0.001
Metastases	58	38	1.94 (1.38–2.73)	<0.001	
**Site of the tumour**					
Other limb site	1562	596	1.00	n/a	<0.001
Proximal femur/humerus	234	124	1.50 (1.22–1.84)	<0.001	
Axial skeleton	71	42	1.53 (1.10–2.13)	0.011	
**WHO classification of sarcoma at diagnosis**					
Conventional: chondroblastic	300	144	1.00	n/a	0.002
Conventional: osteoblastic	1154	484	0.85 (0.71–1.03)	0.101	
Conventional: other	293	99	0.67 (0.52–0.88)	0.003	
Telangiectatic	86	24	0.52 (0.33–0.80)	0.003	
Small cell	10	5	1.48 (0.60–3.64)	0.389	
High-grade surface	24	6	0.44 (0.19–0.99)	0.047	
**Age**					
Child	557	201	1.00	n/a	0.015
Adolescent	921	388	1.25 (1.05–1.48)	0.013	
Adult	389	173	1.32 (1.07–1.63)	0.008	
**Gender**					
Female	761	288	1.00	n/a	0.017
Male	1106	474	1.20 (1.03–1.39)	0.017	
**Relative tumour volume**^b^					
Small (<1/3 of involved bone)	851	307	1.00	n/a	0.002
Large (≥1/3 of involved bone)	680	333	1.29 (1.09–1.51)	0.002	
**Pathological fracture at diagnosis**					
No	1645	669	1.00	n/a	0.966
Yes	222	93	1.00 (0.80–1.26)	0.966	
**Surgical margins achieved**^c^					
Wide/Radical	1593	636	1.00	n/a	0.262
Marginal	249	110	1.03 (0.82–1.30)	0.797	
Intralesional	25	16	1.54 (0.92–2.59)	0.102	

CI, confidence interval; EFS, event-free survival; HR, hazard ratio; WHO, World Health Organisation.

a includes possible metastases.

b 336 missing values imputed.

c as reported by the pathologist.

An additional model with the same patient cohort using overall survival as the outcome demonstrated a similar prognosis impact from each of these factors (Appendix Table 2). The CIs around the estimates are broader for the OS model than those for the EFS model because there are fewer deaths than EFS events.

### 3.3 Outcomes from diagnosis by baseline metastases (‘registration cohort’: N = 2186)

Of the ‘registration cohort’, 1810 of 2186 (83%) of the patients were registered with localised disease (M0), 362 of 2186 (17%) were M1 and metastasis status was not reported for 14 of 2186 (<1%). There were 711 EFS events reported in the M0 patient subset (Table 1). For these M0 patients, 3-year EFS from biopsy was 65% (95% CI: 63–67%), and 5-year EFS from biopsy was 60% (95% CI: 57–62%). With a median follow-up of 56 months, 422 deaths were reported in these patients with localised disease, with 3-year survival from biopsy 84% (95% CI: 82–86%) and 5-year survival from biopsy 76% (95% CI: 74–78%) (Fig. 2B).

For the 362 of 2186 (17%) M1 patients at presentation, the median follow-up was 47 months, and 254 patients reported an EFS event. Three-year EFS from biopsy was 32% (95% CI: 27–37%), and 5-year EFS from biopsy was 28% (95% CI: 23–33%). A total of 194 deaths were reported; 3-year survival from biopsy was 56% (95% CI: 50–61%), and 5-year survival from biopsy was 45% (95% CI: 39–50%) (Fig. 2C).

**Fig. 3:**
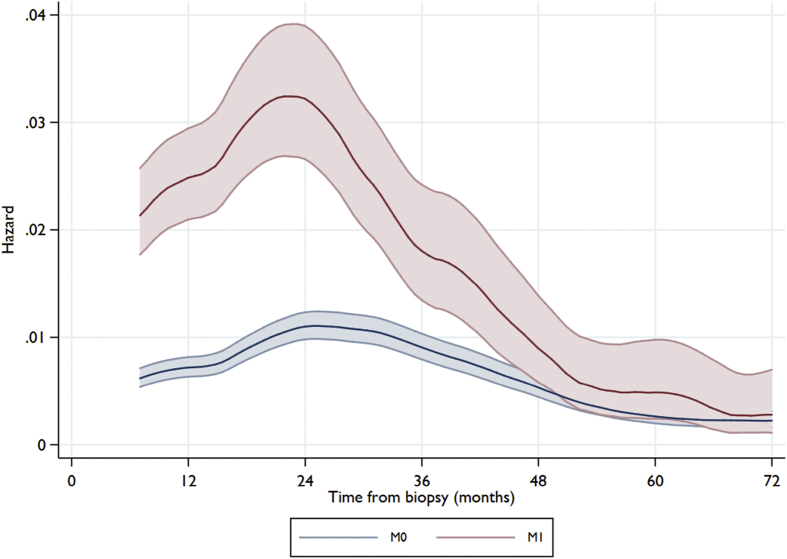
Hazard of event-free survival (EFS) from diagnostic biopsy for all ‘registration cohort’ patients, plotted by metastatic status at registration. **Note:** non-COG patients categorised at registration as having ‘possible’ metastases are included in the M0 category. Shaded area shows 95% CI around estimates. CI, confidence interval; COG, Children's Oncology Group.

The risk of an EFS event was highest around the second year after diagnosis for both M1 and M0 patients. The hazard of event then declines and reaches the same lower level for both M1 and M0 patients four years after diagnosis, but risk continues (Fig. 3).

### 3.4 Outcomes from surgery (‘M0-CSR’ group, N = 1549)

Of the patients registered with localised disease (M0), 1549 of 1810 (86%) M0 patients were considered to have evidence of CSR (‘M0-CSR’ group). These patients had a median follow-up time from surgery of 57 months (IQR: 39-74) with 545 patients reporting an EFS event; 3-year EFS from surgery was 70% (95% CI: 67–72%), and 5-year EFS from surgery was 64% (95% CI: 61–66%). There were 308 deaths reported; 3-year survival from surgery was 88% (95% CI: 86–89%), and 5-year survival from surgery was 79% (95% CI: 77–81%).

In the ‘M0-CSR’ group, 1395 of 1549 (90%) patients were included in the multivariate model of EFS (Table 4); missing tumour volume was imputed for 240 of these patients. Poor histological response to chemotherapy was strongly associated with poorer EFS than a good histological response (HR = 2.13, 95% CI: 1.76–2.58). Poorer EFS was also associated with tumour site on the proximal femur or humerus than other limb site (HR = 1.38, 95% CI: 1.06–1.80) and being adult (HR = 1.53, 95% CI: 1.17–1.99) or adolescent (HR = 1.43, 95% CI: 1.14–1.79) compared to being a child. There was some limited evidence that having a conventional unspecified subtype osteosarcoma classification as opposed to chondroblastic was associated with improved EFS (HR = 0.71, 95% CI: 0.52–0.96); however, pathology overall was not a statistically significant variable in the model (P = 0.157; Table 4). Appendix Table 3 shows survival for the ‘M0-CSR’ group; the interpretation of the prognostic factors is similar to the EFS model for this patient group.

**Table 4:** Cox model for event-free survival (timed from surgery) for all ‘M0-CSR’ patients (*N = 1395)*.

Characteristic	*N*	EFS event*s*	Adjusted HR (95% CI)	*p*-value	Overall *p*-value
**Site of the tumour**					
Other limb site	1175	382	1.00	n/a	0.039
Proximal femur/humerus	166	72	1.38 (1.06–1.80)	0.018	
Axial skeleton	54	27	1.29 (0.86–1.95)	0.214	
**WHO classification of sarcoma at diagnosis**					
Conventional: chondroblastic	240	103	1.00	n/a	0.157
Conventional: osteoblastic	830	284	0.91 (0.72–1.14)	0.408	
Conventional: other	230	69	0.71 (0.52–0.96)	0.029	
Telangiectatic	69	18	0.71 (0.42–1.20)	0.199	
Small cell	6	2	0.79 (0.19–3.20)	0.737	
High-grade surface	20	5	0.45 (0.18–1.12)	0.086	
**Age**					
Child	409	110	1.00	n/a	0.003
Adolescent	689	250	1.43 (1.14–1.79)	0.002	
Adult	297	121	1.53 (1.17–1.99)	0.002	
**Gender**					
Female	586	189	1.00	n/a	0.071
Male	809	292	1.19 (0.99–1.43)	0.071	
**Relative tumour volume**^a^					
Small (<1/3 of the involved bone)	679	214	1.00	n/a	0.046
Large (≥1/3 of the involved bone)	476	197	1.24 (1.00–1.52)	0.046	
**Pathological fracture at diagnosis**					
No	1235	426	1.00	n/a	0.783
Yes	160	55	0.96 (0.71–1.29)	0.783	
**Surgical margins achieved**^b^					
Wide/Radical	1200	403	1.00	n/a	0.201
Marginal	182	71	1.11 (0.83–1.49)	0.482	
Intralesional	13	7	1.98 (0.91–4.30)	0.083	
**Histological response**					
Good (<10% viable tumour)	724	176	1.00	n/a	<0.001
Poor (≥10% viable tumour)	671	305	2.13 (1.76–2.58)	<0.001	

CI, confidence interval; EFS, event-free survival; HR, hazard ratio; WHO, World Health Organisation.

a 240 missing values imputed.

b as reported by the pathologist.

## 4 Discussion

The EURAMOS-1 is the largest osteosarcoma trial performed to date. Of the 2260 patients registered to the protocol, 2186 were eligible for this cohort analysis. The large number of patients and the broad eligibility criteria of patients with operable osteosarcoma, which include patients with axial or metastatic disease, extend the relevance of our findings compared to most other osteosarcoma trials [7], [8], [13], [19], [20].

A three-drug MAP combination, based on COG's INT-0133 trial, was agreed upon as standard therapy for the EURAMOS-1 [7], [8]. Here, the 5-year EFS and survival from diagnosis for all eligible patients were 54% and 71%, respectively. For patients with localised disease, the 5-year EFS (60%) and survival (76%) were comparable to previously reported osteosarcoma studies in patients with tumours entirely or mostly located in extremities, conducted by the founding members of the EURAMOS-1 [7], [8], [13], [20]. Other study groups using different 3- or 4-drug schedules from these same active drugs have reported similar results as the EURAMOS-1 [19], [21]. The patients with metastatic disease recruited to this trial were selected on the condition that the disease was resectable at all sites; 17% of patients were considered to have metastases at diagnosis. In this selected cohort, the reported 5-year EFS from diagnosis of 28% compares well to previous results reported from unselected cohorts of patients with any metastases [22] or patients with only lung metastases [23] but remains unacceptably low. However, comparison to historical data should be made with caution due to the stage shift over the last decades with more patients recorded with primary metastatic disease, probably related to refined imaging techniques. Historically, 10–15% of patients with osteosarcoma are reported to have primary metastatic disease, less than the 17% in this selected cohort excluding patients with deemed non-resectable metastatic disease at presentation [24]. For patients in CSR at all sites (a status achieved 3–6 months after diagnosis), the 5-year EFS and overall survival from biopsy were 64% and 79%, respectively. An important message for patients is that after successful surgery, nearly 4 out of 5 are alive five years from diagnosis and the risk of relapse decreases over time.

The model including prognostic factors available at diagnosis confirmed previously reported results on the impact of metastases, site and tumour size with the strongest impact from the presence of metastases at presentation [10], [11], [14]. Tumour size is a factor of prognostic interest and is likely dependent on many interconnected factors, including the site (which bone) of the tumour and the size of the patient. Relative tumour size was missing for 412 of the patients. We therefore applied a model in which relative tumour size was imputed if missing, based on data available from all otherwise-eligible patients in the models.

Telangiectatic pathology is relatively uncommon among osteosarcoma subtypes [25], here constituting 4.5% of the cases. We observed that the telangiectatic subtype had a more favourable prognosis than osteoblastic osteosarcoma. This has previously been reported in univariate analyses in a small series of 28 patients [26]. Our findings reflect the strength of large series, such as EURAMOS, internationally recruiting many patients which increase the absolute numbers of patients in the series with very rare subgroups, such as telangiectatic osteosarcoma.

We report a statistically significant association between both age and gender on the risk for event. This is in accordance with a meta-analysis including 4838 patients with osteosarcoma in trials and series (not including EURAMOS-1) in which both age and gender were associated with survival, with more favourable outcomes for younger patients and females [16]. For both age groups (child vs adult) and gender, the reported HRs were very similar to the reported values in this study. Thus, a conclusion from these two large series is that there is a significant but modest correlation of both age and gender on survival in osteosarcoma.

Previous attempts to stratify up-front treatment for good (small tumour volume) and poor (metastatic or axial location) prognostic factors have not yet led to improved outcomes [27], [28]. We observed a prognostic impact of histological subtypes (i.e. telangiectatic, high-grade surface and unspecified conventional), consistent with other series but with a different impact from osteosarcoma subtypes on prognosis and in series utilising other chemotherapy regimens [29], [30]. Together, the data suggest biological differences between subtypes; however, prospective trials to test if up-front therapy should be directed by subtype are difficult to conduct because of the rarity of many subtypes.

We performed a prognostic factor analysis adding treatment-related factors. An eligibility criterion for recruitment to the EURAMOS-1 was that surgery with macroscopic clearance was deemed to be possible at all sites. Histologic response was added to the EFS model in addition to the factors at diagnosis. The risk of a subsequent EFS event was more than doubled in patients with poor response to pre-operative chemotherapy. In the COSS report on 1702 consecutive, unselected patients with osteosarcoma including patients with tumour of the extremity and trunk and also patients with metastases at presentation, an HR of 2.4 was reported, similar to the 2.18 in our cohort [10]. Age and tumour site retained their prognostic impact in this model, but there was no good evidence of an impact from gender. Fewer patients were included in this analysis than in the model with all registered patients with localised disease, but the HR reductions, for tumour site (i.e. axial skeleton) and size, probably reflect the more challenging surgery for these tumours and not that the poor prognosis reflect a more aggressive biology.

One limitation of the current report is that it focuses on patients with resectable disease, set up to facilitate recruitment to two specific randomisations. It is likely that those with unresectable disease have a less favourable outlook. Another limitation is missing information on those patients not randomised, which prevented investigation by treatment actually received. To facilitate efforts towards the randomised comparisons and to anticipate that most patients would be randomised, the EURAMOS-1 team did not prospectively collect details of the post-operative phase of treatment, including surgery for metastatic disease and histological response for all these patients. Therefore, there is some selection bias in the models.

The EURAMOS-1 has already demonstrated that large international trials are feasible with no impairment of the quality of care for the patients. Together with the results from the EURAMOS-1 trial based on the randomised patients, we consider the current MAP regimen as a standard chemotherapy in high-grade osteosarcoma in patients aged <40 years, but note that further efforts are required to drive improvements. With the EURAMOS-1 protocol, four collaborating study groups have established a standard for evaluation and treatment of patients with osteosarcoma and a unique platform for further studies; the important matter will be to identify and develop the next appropriate trial.

In conclusion, nearly 4 out of every 5 patients with non-metastatic osteosarcoma who have all disease resected are alive five years later, and the risk of relapse appears to decrease over time. The reported prognostic factors in this large cohort reinforces the impact of known prognostic factors and adds information only achievable from large studies.

## Role of the funding source

The study sponsor was the UK Medical Research Council in Europe and the U.S. National Cancer Institute in North America and Australia. Each trial group organised local coordination elements; central coordination and analysis was led from MRC Clinical Trials Unit at UCL. Neither the sponsors nor the funders of the trial had a role in trial design, data analysis or data interpretation.

The EURAMOS-1 is an academic clinical trial funded through multiple national and international government agencies and cancer charities:

• National Cancer Institute, USA provides funding to the Children's Oncology Group (N America, Australasia and Switzerland) Grant U10CA180886
• European Science Foundation (ESF) under the EUROCORES Program European Clinical Trials (ECT), through contract No. ERASCT-2003-980409 of the European Commission, DG Research, FP6 (Ref No MM/NG/EMRC/0202)
• National funding in Europe:
  • Belgium: FNRS (Fonds National de la Recherche Scientifique) BelgiumFWO (Fonds voor Wetenschappelijk Onderzoek-Vlaanderen
  • Denmark: Danish Medical Research Council
  • Finland: Academy of Finland
  • Germany: DFG ref No: BI 1045/1-1 & 1–2, DKH ref No: 50-2723-Bi2
  • Hungary: Semelweis Foundation
  • Netherlands: ZonMw (Council for Medical Research)
  • Norway: Research Council of Norway
  • Sweden: Scandinavian Sarcoma Group
  • Switzerland: Swiss Paediatric Oncology Group (SPOG)
  • United Kingdom: includes funding for the trial co-ordinating data centre (MRC Clinical Trials Unit at UCL): Cancer Research UK, CRUK/05/013, Medical Research Council: MC_UU_12023/28

Additional funding to the University of Muenster Centre for Clinical Trials, site of the EURAMOS Intergroup Safety Desk: Federal Ministry of Education and Research, Germany, BMBF 01KN1105.

## Conflict of interest statement

The following authors report the following possible conflicts of interest. All others have none to report.

Dr. Bielack reports grants from Deutsche Krebshilfe, Deutsche Forschungsgemeinschaft and European Science Foundation during the conduct of the study and personal fees from Lilly, Bayer, Pfizer, Novartis, Isofol and Clinigen, outside the submitted work. P.R. reports grants and personal fees from Novartis and personal fees from Pfizer, Bayer, PharmaMar, Amgen, AstraZeneca, Clinigen, Lilly and Deciphera, outside the submitted work. Dr Teot reports work under consideration for publication and COG subcontractor fees for pathology review, outside the submitted work. Dr Gebhardt reports other support from Clinical Orthopaedics and Related Research and other fees from Up-to-date, outside of the submitted work. Dr Meyers reports stock or other ownership in Amgen, Bayer, Dupont, Henry Schein, Jazz Pharmaceuticals, Mednax, Novartis, Procter and Gamble and Sigma–Aldrich; honoraria from France Foundation and personal fees from Takeda Pharmaceuticals, Medison and InterMune, outside the submitted work. Dr. Lessnick serves as a member of the Scientific Advisory Board for Salarius Pharmaceuticals, a company involved in cancer therapy development outside of the submitted work. Dr. Butterfass-Bahloul reports grants from the Federal Ministry of Education and Research, Germany, other support from European Science Foundation (ESF) under the EUROCORES Program European Clinical Trials (ECT), other from Deutsche Forschungsgemeinschaft (DFG), during the conduct of the study. Dr. Marina reports other from Five Prime Therapeutics, outside the submitted work.

## Figures and Tables

**Appendix Table A1:** Cox model for event-free survival (timed from diagnostic biopsy) for ‘registration cohort’ patients (*N = 1531*)—complete-case analysis with no imputed data.

Characteristic	*N*	EFS events	Adjusted HR (95% CI)	*p*-value	Overall *p-*value
**Pulmonary metastases**					
No metastases^a^	1349	516	1.00	n/a	<0.001
Metastases	182	124	2.39 (1.96–2.93)	<0.001	
**Non-pulmonary metastases**					
No metastases^a^	1485	609	1.00	n/a	<0.001
Metastases	46	31	1.97 (1.35–2.88)	<0.001	
**Site of the tumour**					
Other limb site	1276	499	1.00	n/a	<0.001
Proximal femur/humerus	199	105	1.42 (1.13–1.77)	<0.001	
Axial skeleton	56	36	1.59 (1.12–2.28)	0.010	
**WHO classification of sarcoma at diagnosis**					
Conventional: chondroblastic	252	123	1.00	n/a	<0.001
Conventional: osteoblastic	943	403	0.89 (0.72–1.09)	0.256	
Conventional: other	235	82	0.71 (0.54–0.95)	0.021	
Telangiectatic	74	23	0.58 (0.37–0.91)	0.017	
Small cell	7	3	1.15 (0.36–3.65)	0.811	
High-grade surface	20	6	0.56 (0.25–1.29)	0.171	
**Age**					
Child	466	172	1.00	n/a	0.004
Adolescent	742	323	1.29 (1.07–1.56)	0.008	
Adult	323	145	1.33 (1.06–1.66)	0.015	
**Gender**					
Female	639	251	1.00	n/a	0.015
Male	892	389	1.18 (1.01–1.39)	0.043	
**Relative tumour volume**^b^					
Small (<1/3 of involved bone)	851	307	1.00	n/a	<0.001
Large (≥1/3 of involved bone)	680	333	1.35 (1.14–1.59)	<0.001	
**Pathological fracture at diagnosis**					
No	1343	558	1.00	n/a	0.457
Yes	188	82	1.00 (0.79–1.28)	0.992	
**Surgical margins achieved**^c^					
Wide/Radical	1295	532	1.00	n/a	0.019
Marginal	214	94	1.02 (0.79–1.31)	0.872	
Intralesional	22	14	1.41 (0.81–2.46)	0.226	

CI, confidence interval; EFS, event-free survival; HR, hazard ratio; WHO, World Health Organisation.

a includes possible metastases.

b as reported with no imputed data.

c as reported by the pathologist. **Note:** 336 missing values on relative tumour volume not imputed (as shown in Table 3 in main text, for comparison).

**Appendix Table A2:** Cox model for overall survival (timed from diagnostic biopsy) for ‘registration cohort’ patients (*N = 1867*).

Characteristic	*N*	OS events	Adjusted HR (95% CI)	*p*-value	Overall *p*-value
**Pulmonary metastases**					
No metastases^a^	1633	368	1.00	n/a	<0.001
Metastases	234	110	2.25 (1.80–2.82)	<0.001	
**Other metastases**					
No metastases^a^	1809	444	1.00	n/a	<0.001
Metastases	58	34	2.79 (1.92–4.04)	<0.001	
**Site of the tumour**					
Other limb site	1562	360	1.00	n/a	<0.001
Proximal femur/humerus	234	86	1.67 (1.30–2.14)	<0.001	
Axial skeleton	71	32	1.85 (1.25–2.72)	0.002	
**WHO classification of sarcoma at diagnosis**					
Conventional: chondroblastic	300	87	1.00	n/a	0.012
Conventional: osteoblastic	1154	314	0.91 (0.72–1.16)	0.469	
Conventional: other	293	57	0.66 (0.47–0.93)	0.016	
Telangiectatic	86	14	0.49 (0.28–0.87)	0.015	
Small cell	10	4	1.47 (0.53–4.06)	0.457	
High-grade surface	24	2	0.28 (0.07–1.14)	0.076	
**Age**					
Child	557	123	1.00	n/a	0.044
Adolescent	921	250	1.32 (1.06–1.65)	0.014	
Adult	389	105	1.27 (0.97–1.66)	0.081	
**Gender**					
Female	761	163	1.00	n/a	0.001
Male	1106	315	1.40 (1.16–1.70)	0.001	
**Relative tumour volume**^b^					
Small (<1/3 of involved bone)	851	191	1.00	n/a	0.063
Large (≥1/3 of involved bone)	680	211	1.21 (0.99–1.49)	0.063	
**Pathological fracture at diagnosis**					
No	1645	417	1.00	n/a	0.612
Yes	222	61	1.08 (0.81–1.42)	0.612	
**Surgical margins achieved**^c^					
Wide/Radical	1593	394	1.00	n/a	0.036
Marginal	249	70	0.93 (0.69–1.26)	0.652	
Intralesional	25	14	2.00 (1.13–3.52)	0.017	

CI, confidence interval; EFS, event-free survival; HR, hazard ratio; WHO, World Health Organisation.

a includes possible metastases.

b 336 missing values imputed.

c as reported by the pathologist.

**Appendix Table A3:** Cox model for overall survival (timed from surgery) for ‘M0-CSR’ patients (*N = 1395*).

Characteristic	*N*	OS event*s*	Adjusted HR (95% CI)	*p*-value	Overall *p*-value
**Site of the tumour**					
Other limb site	1175	200	1.00	n/a	0.001
Proximal femur/humerus					
	166	47	1.76 (1.25–2.48)	0.001	
Axial skeleton	54	20	1.74 (1.06–2.85)	0.027	
**WHO classification of sarcoma at diagnosis**					
Conventional: chondroblastic	240	59	1.00	n/a	0.141
Conventional: osteoblastic	830	159	0.90 (0.67–1.23)	0.514	
Conventional: other	230	37	0.66 (0.43–1.01)	0.054	
Telangiectatic	69	9	0.57 (0.27–1.19)	0.136	
Small cell	6	2	1.42 (0.34–5.83)	0.629	
High-grade surface	20	1	0.18 (0.02–1.28)	0.086	
**Age**					
Child	409	59	1.00	n/a	0.041
Adolescent	689	147	1.48 (1.09–2.01)	0.012	
Adult	297	61	1.26 (0.87–1.84)	0.217	
**Gender**					
Female	586	90	1.00	n/a	0.001
Male	809	177	1.52 (1.18–1.97)	0.001	
**Relative tumour volume**^a^					
Small (<1/3 of the involved bone)	679	116	1.00	n/a	0.185
Large (≥1/3 of the involved bone)	476	113	1.19 (0.92–1.55)	0.185	
**Pathological fracture at diagnosis**					
No	1235	234	1.00	n/a	0.851
Yes	160	33	1.04 (0.70–1.53)	0.851	
**Surgical margins achieved**^b^					
Wide/Radical	1200	218	1.00	n/a	0.073
Marginal	182	43	1.13 (0.77–1.65)	0.533	
Intralesional	13	6	2.73 (1.15–6.47)	0.023	
**Histological response**					
Good (<10% viable tumour)	724	87	1.00	n/a	<0.001
Poor (≥10% viable tumour)	671	180	2.45 (1.88–3.20)	<0.001	

CI, confidence interval; EFS, event-free survival; HR, hazard ratio; WHO, World Health Organisation.

a 240 missing values imputed.

b as reported by the pathologist.
